# Tolerance of Bupropion SR After Delayed-Onset Urticaria and Angioedema Associated With Bupropion XL

**DOI:** 10.1155/2024/6638911

**Published:** 2024-10-23

**Authors:** Faisal R. Elali, Arthur C. Grant

**Affiliations:** ^1^College of Medicine, SUNY Downstate Health Sciences University, 450 Clarkson Avenue, Brooklyn 11203, New York, USA; ^2^Department of Neurology, SUNY Downstate Health Sciences University, 450 Clarkson Avenue., Brooklyn 11203, New York, USA

## Abstract

Bupropion is an atypical antidepressant indicated for the treatment of major depressive disorder (MDD), seasonal affective disorder (SAD), and smoking cessation. It is also used off-label for attention deficit hyperactivity disorder (ADHD). Its mechanism of action includes the selective norepinephrine and dopamine reuptake inhibitor (NDRI). The drug is available in immediate-release (IR), sustained-release (SR), and extended-release (XL) formulations. Common side effects are typically mild and include anxiety, insomnia, headache, dizziness, constipation, and nausea. Rarely, cutaneous hypersensitivity reactions may occur. We describe a 23-year-old man who developed severe and diffuse urticaria and angioedema 4 weeks after initiation of bupropion XL for MDD and ADHD. The bupropion was stopped, and he was treated with levocetirizine, diphenhydramine (oral and topical), and methylprednisolone with complete resolution of his symptoms within 2 weeks. Due to a good initial therapeutic response to the medication, a trial of bupropion SR was initiated. The patient again had a favorable therapeutic response without any dermatologic side effects.

## 1. Introduction

Major depressive disorder (MDD) is a common disease, with an incidence of 8.4% and prevalence of 17.0% in adults in the United States [1]. MDD is associated with significant morbidity and mortality, often due to suicide [2]. Selective serotonin reuptake inhibitors (SSRIs) and serotonin and norepinephrine reuptake inhibitors (SNRIs) are widely used as first-line MDD treatment options. Bupropion, a selective norepinephrine and dopamine reuptake inhibitor (NDRI), is an atypical antidepressant with a unique mechanism of action among antidepressants [3]. It is indicated for the treatment of MDD, seasonal affective disorder (SAD), and smoking cessation (under the brand name Zyban). Additionally, it is used off-label for attention deficit hyperactivity disorder (ADHD), among other conditions. Common side effects are usually mild and include anxiety, insomnia, headache, dizziness, constipation, and nausea [4]. Convulsive seizures are a well-established dose-related risk, with an incidence of 0.4% in patients taking up to 450 mg per day [5]. Hypersensitivity reactions are rare, with an estimated incidence of roughly 1% [6].

## 2. Case Presentation

A 23-year-old man was diagnosed with MDD, ADHD, posttraumatic stress disorder (PTSD), and binge-eating disorder. He had been experiencing low mood, anhedonia, difficulty concentrating, hyperactivity, and binge-eating episodes for the past 2 years, worsening in the past 6 months, which he attributes to his role as a student in graduate school. He reported that his symptoms had begun impairing his academic performance, social relationships, and daily functioning. According to his family doctor, his last Patient Health Questionnaire-9 (PHQ-9) was given roughly 1 year prior to his initial presentation, scoring 17/27, falling under moderately severe depression. He was told to follow-up with a psychiatrist but delayed doing so as he was currently going through the graduate school admission process and it was not a priority for him, at the time. He then began seeing his school-provided psychiatrist, who began treatment for MDD and ADHD.

Mental status examination revealed several findings. He was well-groomed and dressed appropriately during examination. He was cooperative and pleasant to speak with. There were no psychomotor changes, specifically in movement, position, or posture. He spoke with a normal rate, rhythm, volume, and prosody. Depressed mood with congruent affect was noted. His thought process was coherent but somewhat slowed, with slight impairment in attention. His thought content was unremarkable, future oriented, and nonpsychotic. He had no suicide, homicide, or violent ideation toward himself or others. His insight and judgement were intact.

**Figure 1:**
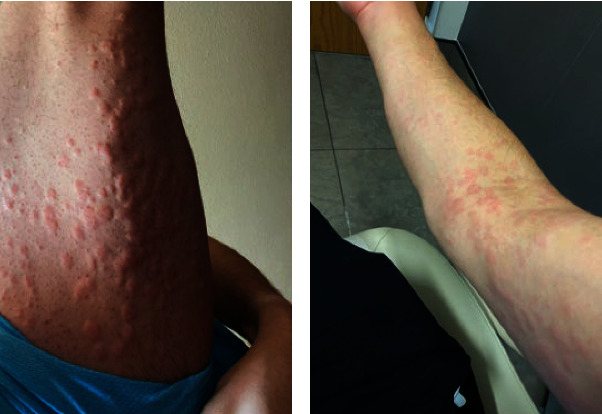
Left posterior thigh (a) and anterior–medial right arm and forearm (b) showing diffuse and confluent erythematous wheals. There were similar lesions on his face, trunk, back, and other extremities.

**Figure 2:**
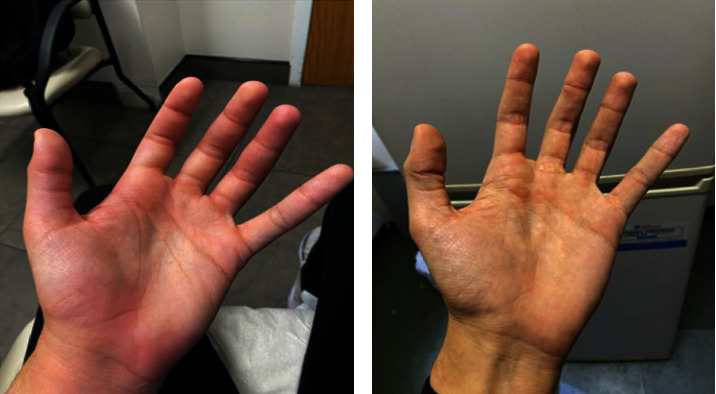
Angioedema and erythema of the palmar side of the left hand (a) with complete resolution 2 weeks later (b).

He was treated with cognitive behavioral therapy and bupropion XL 150 mg daily for his MDD and ADHD, escalating to 300 mg per day after 2 weeks as he was demonstrating improvement in his overall depressive and attention-related symptoms. The patient had a remarkable initial response to the medication, particularly with respect to the ADHD symptoms. Unfortunately, 28 days after initiation of bupropion, he woke up in the morning and found that he developed highly pruritic, diffuse, and confluent erythematous wheals over his entire body (Figure 1) and angioedema on the palmar side of his hands (Figure 2). He was unable to use his hands because of the remarkable swelling, further impairing his daily functioning as a graduate student. There was no sign of airway involvement, and the remainder of his physical examination was unremarkable. He had no known allergies and no history of cutaneous drug reactions. There were no changes to his diet, environment, soap, or detergents.

The only recent change in his life was the bupropion, which was immediately discontinued, with levocetirizine 5 mg per day and diphenhydramine 25 mg per day orally (and topically as needed) being initiated. Five days later, there was significant improvement of the rash but only moderate improvement of the angioedema, and a 1-week course of methylprednisolone 4 mg per day was initiated. Two weeks after initial onset of his signs and symptoms, the rash and angioedema had completely resolved.

Because the patient initially had a beneficial clinical response to bupropion XL, he and his psychiatrist agreed to a trial of bupropion SR 150 mg twice a day. At his last 3- and 12-month follow-ups, he remained free of cutaneous reactions and had substantial improvement in his MDD and ADHD symptoms.

## 3. Discussion

Severe dermatologic reactions to bupropion have been described, including urticaria, angioedema, exanthematous pustulosis, and others [7–10]. The incidence of urticaria associated with bupropion is ~1%, with men under age 40 having a higher incidence than other populations [8]. Hu et al. performed a nationwide cohort study of patients taking bupropion from 2000 to 2009 and found that the risk of developing urticaria within the first 4 weeks of treatment was higher than any time after 4 weeks (risk ratio 1.84; 95%CI, 1.28–2.54; *p* = 0.001). Within the first 4 weeks of treatment, urticaria was more common in weeks 3 and 4 compared to weeks 1 and 2 (*p* = 0.002) [8]. As is true for most drugs that trigger cutaneous hypersensitivity reactions, the precise molecular mechanism of urticaria and angioedema due to bupropion is not well understood. One possibility is its structural similarity to amfepramone, a norepinephrine-releasing drug associated with adrenergic urticaria due to stress [11, 12].

Treatment of the urticaria and angioedema begins with discontinuing the bupropion and initiating a short course of oral antihistamines (e.g., cetirizine, levocetirizine, and loratadine) and in severe cases oral corticosteroids (e.g., prednisone and methylprednisolone). Topical antihistamines (e.g., diphenhydramine hydrochloride) and high-potency topical steroids (e.g., triamcinolone, fluocinonide, clobetasol, and halobetasol) may also be used.

Once hypersensitivity-related signs and symptoms have resolved, the patient and their physician must choose a treatment plan that balances anticipated benefit and risk. In most cases, this will mean switching to a different medication or possibly even a different class of medication. However, as this case illustrates, when the therapeutic benefit of the offending agent is high, reintroduction of the same drug should not be excluded as a viable option. Naturally, the patient should remain vigilant to the signs and symptoms of cutaneous or other hypersensitivity reactions and told to stop the medication and contact their physician at the first sign of an allergic reaction. In this case, the patient had a good therapeutic response without a hypersensitivity reaction to the bupropion SR formulation at the same dose as the bupropion XL that triggered the reaction.

Since he tolerated bupropion SR, it is possible that the patient was allergic to an inactive ingredient in bupropion XL, but we think this unlikely. First, severe dermatologic reactions to bupropion are well-established in the medical literature [7–12]. Second, the inactive ingredients are very common in medications and foodstuffs, and it is highly likely that he was exposed to these substances earlier in life (Table 1). Third, according to the Naranjo et al. [15] Adverse Drug Reaction Probability Scale, the association between bupropion and this patient's dermatologic reactions is in the “probable” category (Table 2) [15]. Fourth, the release mechanism between the XL and SR formulations differ [16]. The bupropion maximum serum concentration is slightly higher with XL formulation taken once a day compared to the SR formulation taken twice a day. Finally, there are well-documented examples (e.g., lamotrigine) of drugs that caused cutaneous hypersensitivity reactions being successfully reintroduced with a longer titration phase or different formulation [17, 18].

## 4. Conclusion

Bupropion may produce delayed-onset urticaria and angioedema that respond well to drug cessation and a short course of antihistamines and steroids. In patients for whom the drug is of unambiguous clinical benefit, a cutaneous hypersensitivity reaction is not an absolute contraindication to a trial of a different formulation of the medication, as shown in this case.

## Figures and Tables

**Table 1:** Active, inactive, and printing ingredients in bupropion XL and bupropion SR [13, 14].

Medication name	Bupropion XL	Bupropion SR
Active ingredient	Bupropion hydrochloride	Bupropion hydrochloride

Inactive ingredients	Polyethylene glycol Ethylcellulose aqueous dispersion Glyceryl behenate Methacrylic acid copolymer dispersion Polyvinyl alcohol Povidone Silicon dioxide Triethyl citrate	Polyethylene glycol Carnauba wax Cysteine hydrochloride Hypromellose Magnesium stearate Microcrystalline cellulose Polysorbate 80 Titanium dioxide

Printing ingredient	Edible black ink	Edible black ink

**Table 2:** Naranjo Scale Questionnaire estimates the probable association between a drug and a putative adverse reaction.

Question	Yes	No	Unknown	Patient's score
Are there previous conclusive reports of this reaction?	+1	0	0	+1
Did the adverse event appear after the drug was given?	+2	−1	0	+2
Did the adverse reaction improve when the drug was discontinued or a specific antagonist was given?	+1	0	0	+1
Did the adverse reaction reappear upon readministering the drug?	+2	−1	0	−1
Were there other possible causes for the reaction?	−1	+2	0	+2
Did the adverse reaction reappear upon administration of placebo?	−1	+1	0	0
Was the drug detected in the blood or other fluids in toxic concentrations?	+1	0	0	0
Was the reaction worsened upon increasing the dose? Or was the reaction lessened upon decreasing the dose?	+1	0	0	+1
Did the patient have a similar reaction to the drug or a related agent in the past?	+1	0	0	0
Was the adverse event confirmed by any other objective evidence?	+1	0	0	+1
Total score	—	—	—	**7**

*Note:* The scales are as follows: doubtful (≤0), possible (1–4), probable (5–8), and definitive (≥9). Our patient's score is 7, corresponding to the probable category [15]. The bold value indicates the patient's condition in relation to how probable the association between the drug the patient took and a putative adverse reaction occurring.

## Data Availability

The data that support the findings of this study are available on request from the corresponding author. The data are not publicly available due to privacy or ethical restrictions.
